# Yin Yang 1: Function, Mechanisms, and Glia

**DOI:** 10.1007/s11064-025-04345-7

**Published:** 2025-02-04

**Authors:** Ada G. Rodríguez-Campuzano, Francisco Castelán, Luisa C. Hernández-Kelly, Marie-Paule Felder-Schmittbuhl, Arturo Ortega

**Affiliations:** 1 Departamento de Biología Celular y Fisiología, Unidad Foránea Tlaxcala, Instituto de Investigaciones Biomédicas, Universidad Nacional Autónoma de México, Tlaxcala, Tlaxcala Mexico; 2 Departamento de Toxicología, Centro de Investigación y de Estudios Avanzados del Instituto Politécnico Nacional, Av. IPN 2508, San Pedro Zacantenco, G.A. Madero, 07360 Ciudad de Mexico, Mexico; 3 Centre National de la Recherche Scientifique, Institut des Neurosciences Cellulaires et Intégratives (UPR 3212), Université de Strasbourg, Strasbourg, France

**Keywords:** Yin Yang 1, Transcription mechanisms, Cell signaling, Glia cells

## Abstract

Yin Yang 1 is a ubiquitously expressed transcription factor that has been extensively studied given its particular dual transcriptional regulation. Yin Yang 1 is involved in various cellular processes like cell cycle progression, cell differentiation, DNA repair, cell survival and apoptosis among others. Its malfunction or alteration leads to disease and even to malignant transformation. This transcription factor is essential for the proper central nervous system development and function. The activity of Yin Yang 1 depends on its interacting partners, promoter environment and chromatin structure, however, its mechanistic activity is not completely understood. In this review, we briefly discuss the Yin Yang 1 structure, post-translational modifications, interactions, mechanistic functions and its participation in neurodevelopment. We also discuss its expression and critical involvement in the physiology and physiopathology of glial cells, summarizing the contribution of Yin Yang 1 on different aspects of cellular function.

## Introduction

### Yin Yang 1: A Multifaceted Protein

Yin Yang 1 (YY1) is an ubiquitously distributed transcription factor (TF) in vertebrate cells, that is involved in multiple cellular processes, including development [1], cell growth [2], proliferation [3], differentiation [4], cell cycle [2], DNA repair [5] and apoptosis [6]. YY1 pleiotropic characteristics make it an important participant in tumorigenic processes, hence the necessity of understanding its functions [7]. YY1 was first cloned and described in 1991 simultaneously by various research groups that gave different names to the same protein, those names were based upon the cellular context and molecular mechanisms associated to that protein, it was referred as NF-E1 [8], Delta (δ) [9] and based on its dual activity as transcriptional activator or repressor, Shi et al*.,* named it YY1 [10]. Later on, other authors assigned YY1 other denominations like Upstream Conserved Region Binding Protein (UCRBP) [11], nuclear matrix protein NMP1 [12] and common factor 1 [13]. YY1 multifaceted characteristic is due to its capability of acting as a transcriptional repressor, activator, or an element binding protein.

Over the past two decades, many studies have identified a myriad of genes targeted by YY1, those genes contain at least one of the two most frequent core binding elements for this transcription factor: CCAT and ACAT [14, 15]. Being 5’-CCG[C/A]CATNTT-3’ the small motif to which YY1 binds and that is often found in enhancers and promoters [16]. However, the specific cues dictating when and how the transcriptional decision of YY1 is executed remains elusive. There are some features that may determine the specific final effect of YY1 on gene expression, like the promoter contextual (sequences surrounding the binding sites), chromatin structure, co-interaction with a specific set of proteins, cellular localization, protein relative concentration, cleavage and posttranslational modifications [17–19]. Nowadays, due to all this evidence YY1 is considered a regulator of global gene expression.

In this contribution, we provide, first an overview of YY1 mechanistical and functional properties, and thereafter its role in transcriptional control and its relevance to the Central Nervous System (CNS). Special focus was placed to YY1 in glial cells and how it impacts the CNS physiology at molecular and cellular levels.

### Transcriptional Regulation of YY1

The human YY1 gene is located in chromosome 14 segment q32.2 near the telomere region, in over 40 270 base pairs (bp) in length, and it is encoded in five exons [20]. YY1 pre-mRNA can be spliced into a mature mRNA containing 3159 nucleotides (nt) (480 nt for its 5’-UTR, 1434 nt to the 3’-UTR and 1245 bp of coding sequence)[21]. YY1 is ubiquitously expressed regardless the cell cycle stage or the differentiation status of the cell and has a half-life of about 3.5–4 h [22]. Although, given the plethora of cellular mechanisms regulated by YY1, the *cis-* and *trans-*acting regulatory factors that modulate its own transcription, have not been extensively studied. The transcriptional control of YY1 is under the specificity protein 1 (Sp1) TF, the YY1 promoter region contains multiple Sp1-binding sites (− 57, + 29 and + 231 bp), interestingly it does not contains neither TATA nor CCAAT box, classifying YY1 as a constitutively expressed housekeeping gene [20]. Moreover, the YY1 promoter contains an upstream conserved consensus binding site for CREB/ATF (− 142 bp) and three tandem Myb binding sites (− 736 to − 673 bp). The critical elements for its basal transcription (minimal promoter) reside very near the transcription start site, being sufficient for its transcriptional responsiveness [20]. The NF-κB (p65(RelA)/p50) complex can bind to YY1 proximal promoter and activate its transcription [23, 24]. Ultimately, YY1 can also be regulated by itself, in a negative feed-loop, YY1 is capable to bind to its own DNA-binding sites present within the first intron and by these means downregulate its own mRNA-protein levels [25].

More recently, YY1 expression has been attributed to epigenetic mechanisms rather than direct genomic mechanisms, and most of these mechanisms are associated to carcinogenic events. For instance, taking into consideration its long 3’-UTR, YY1 mRNA is the target of several micro-RNAs (miRNAs) that diminish its translation, for example, miR-7 [26], miR-29a [27], miR-181 [28], miRNA-141-3p [29], miR-98-3p [30], miR-544 [31], miR-186 [32], miR-218 [33], among others [34]. Conversely, long non-coding RNAs (lncRNAs) can promote YY1 mRNA translation, like NPCCAT1 that binds to the 5’-UTR of YY1 mRNA and upregulates its translation [35]. In any event, the mechanisms regulating YY1 expression are by no means simplistic, for example, it has been documented that regulation initiates with lncRNAs that upregulate, downregulate or act as competing endogenous RNA (ceRNA) of the expression/function of miRNAs that target YY1, like the lncRNA LINC00899 whose downstream gene is the miR-744-3p which directly interacts with YY1 mRNA decreasing its expression [36], or the lnc-TLCD2-1 that promotes YY1 expression by targeting and blocking the miR-193A-5p [37]. The lnc-HOTAIR functions as a ceRNA and sponges miR-1 and miR-206 promoting YY1 expression [38]. Another ceRNA is LINC00958 and sponges the miR-378-3p that targets YY1 mRNA, therefore increases its expression [39]. On top of that, circular RNAs, like circ-AGFG1 has been found to sponge miR-4262 and miR-185-5p, upregulating YY1 expression [40]. Much of the current research of YY1 expression is aimed to deeply understand the role of this TF in several regulatory processes in health and disease.

### YY1 Structure

**Fig. 1:**
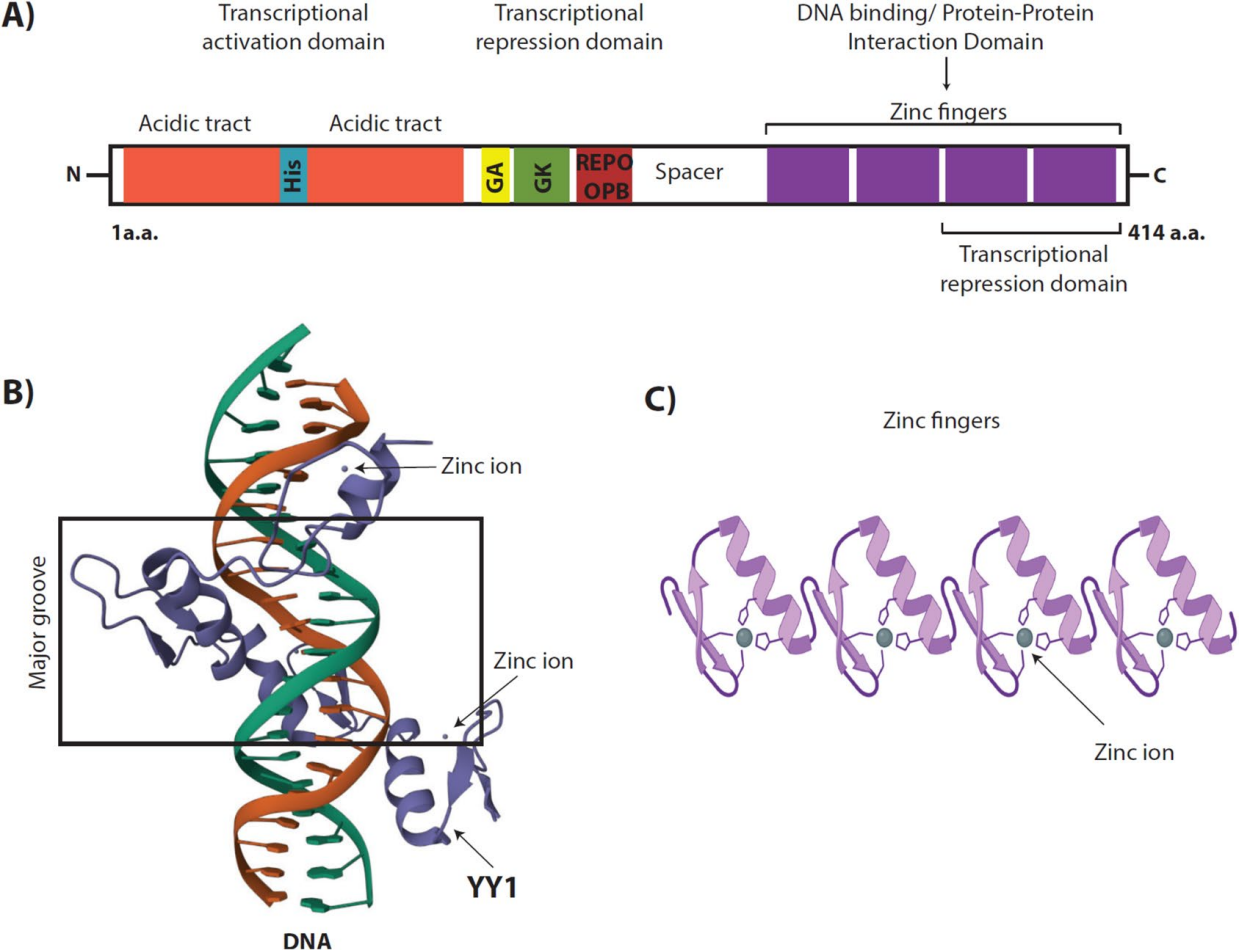
YY1 Diagram of domains and structure. **A** Diagram corresponding to the 414 amino acids of YY1 protein, domains are indicated with different colors starting from the N-terminal that mediates the transcriptional activation mechanisms, passing through the middle of the protein where important domains as the REPO/OPB and GK domains mediate transcriptional repression, and ending in the C-termini containing the four C2H2-type zinc fingers in charge of its DNA binding ability and protein–protein interactions, but also account for a transcriptional repression domain. **B** 3D structure of human YY1 zin finger domain (purple ribbon) bound to the Adeno-Associated Virus P5 initiator element (DNA chains in orange and green) PDB DOI: https://doi.org/10.2210/pdb1UBD/pdb [42] **C** Four serial C2H2 zinc finger motifs (2 Cys and 2 His residues bonded tetrahedrally to Zn ion) consisting each in a short antiparallel β-sheet formed by two strands and hairpin turn followed by an α-helix [43]

YY1 contains 414 amino acids resulting in a 44.713 kDa protein, however in SDS–polyacrylamide gels YY1 migrates as a 65–68 kDa protein, probably due to post-translational modifications [41] (See 1.5 section). YY1 belongs to the GLI-Krüppel class of zinc finger TFs, in the C-terminus it contains four C_2_H_2_-type zinc fingers (amino acids 298–397) that are fundamental for its DNA-binding capability (Fig. 1A) [9, 42]. A zinc finger is a small peptide domain, of around 25 to 30 amino acid residues, that contains a zinc ion tetrahedrally coordinated with Cys and His residues (C_2_H_2_) stabilizing a secondary structure of two β-pleaded sheets in the amino terminal half and an α-helix in the carboxyl terminal half [43]. Many zinc fingers can be continuously aligned with one another fitting in the major groove of DNA (Fig. 1B, C) [44]. However, nowadays it is well established that the zinc-finger motifs can also play important protein–protein interactions modulating the actual regulatory mechanism to be executed on DNA-binding specificity basis [45].

According to site-directed mutagenesis studies, the transcriptional activation of YY1 is located in two domains embedded in the amino terminus within the amino acids (aa) 16–29 and aa 80–100 for maximal activity, whereas the repressive mechanism lies within the zing finger region near the carboxyl terminus between aa 298 and 397, particularly the region belonging to zinc fingers 3 and 4 (aa 333–397), although the structure of this region is not strongly necessary for the repression activity [17, 46]. In the central part of the protein, from aa 154 to 201, YY1 contains a high percentage (55%) of glycine-alanine (GA) and glycine-lysine (GK) rich domains, that also possess transcriptional repression activity [17, 41]. From aa 201 to 226 there is a domain that was named REPO domain giving its ability to REcruit POlycomb group proteins to DNA[47, 48], but more recently it has been re-named as OncoProtein Binding (OPB) domain indispensable for oligomerization and through which YY1 interacts with oncoproteins [49]. Its nuclear targeting domain appears to be located in the C-terminal domain ranging from the aa 257 to 341 [50], including zinc fingers 2 and 3 [22].

In the N-terminal domain, YY1 contains two acidic tracts, beginning with an acidic amphipathic helix (residues 16–29), embedded in the activation domain (aa 43–53) 11 consecutive acidic amino acids (glutamic/aspartic acids), and a stretch rich in glycine followed by a homo-polymeric region of 11 consecutive histidines (aa 70–80) and a region rich in glutamine and proline (aa 81–100) [17] (Fig. 1a). The histidine track appears to modulate its nuclear localization directing YY1 to nuclear speckles resalting a potential role of this protein on RNA metabolism [51, 52]. Moreover, the YY1 histidine track has been recognized to form a liquid–liquid phase separation that enables a compartmentalization together with incorporation of major co-activators and stabilization by distal enhancers to activate target gene expression [53]. Given the nature of the N-terminal of YY1, it may employ electrostatic forces to interact with other proteins, particularly those with positive charges as histones [54]. More recently, it has been found that YY1 N-terminal domain is intrinsically-disordered [55], but under the influence of zinc ions (3 to 5 additional zinc ions more than the 4 already needed in its zinc fingers domains), YY1 can be modulated both in structure and activity, acquiring a more organized structure by modulating its conformational dynamics and restricting its flexibility, this facilitates its dimerization and hence its activity [56, 57].

Some studies have shown that YY1 binds itself to DNA sequences with low affinity and specificity, and that it can achieve higher specificity by associating with co-regulators or as part of multi-subunit complexes [58], while, recent evidence suggests that YY1-DNA interactions are stronger in the presence of tandem YY1 binding sites with no need of additional protein partners [59]. However, YY1 tends to dimerize to promote interactions with distant recognitions sequences bending and looping DNA [60, 61]. Moreover, some studies have suggested that YY1 possess an oligomeric structure, allowing its binding to other proteins [49, 62]. Also, YY1 can bind strongly to RNA particles [63, 64].

### YY1 Subcellular Localization

In accordance with its function YY1 is primarily a nuclear protein, however, it has a mosaic pattern of nucleocytoplasmic distribution [1, 65]. Once in the nucleus, YY1 can be found associated in the nuclear matrix in a high-molecular-weight complex [46], when it is not positioned on DNA elements.

YY1 subcellular localization changes throughout development [66], cell cycle [67] and differentiation [1] proving its relevance in such important cellular processes and the complex regulation YY1 exerts in the cells. In oocytes, YY1 is associated to cytoplasmic messenger ribonucleoprotein particles (mRNPs) participating in the storage and stabilization of maternally transcribed mRNAs required for subsequent embryonic development [63, 64]. During cell cycle, YY1 fluctuates from mainly cytoplasmic at G1 to mainly nuclear at early and middle S phase, and later in S phase it returns to the cytoplasm, moreover, in the early S phase, its DNA-binding activity increases dramatically with no changes in protein and mRNA levels [67]. While, during mitosis its localization also changes, remaining distributed in the cytoplasm during prophase and remains excluded from DNA until early telophase [68]. It has been observed that under apoptosis induction, YY1 changes its localization rapidly from the cytoplasm to the nucleus, hypothesizing that some signals of the cellular machinery regulating DNA status can modulate YY1 subcellular localization [69] and, that such localization is dependent on actin polymerization [70].

### YY1 Posttranslational Modifications and Functional Outcomes

**Fig. 2:**
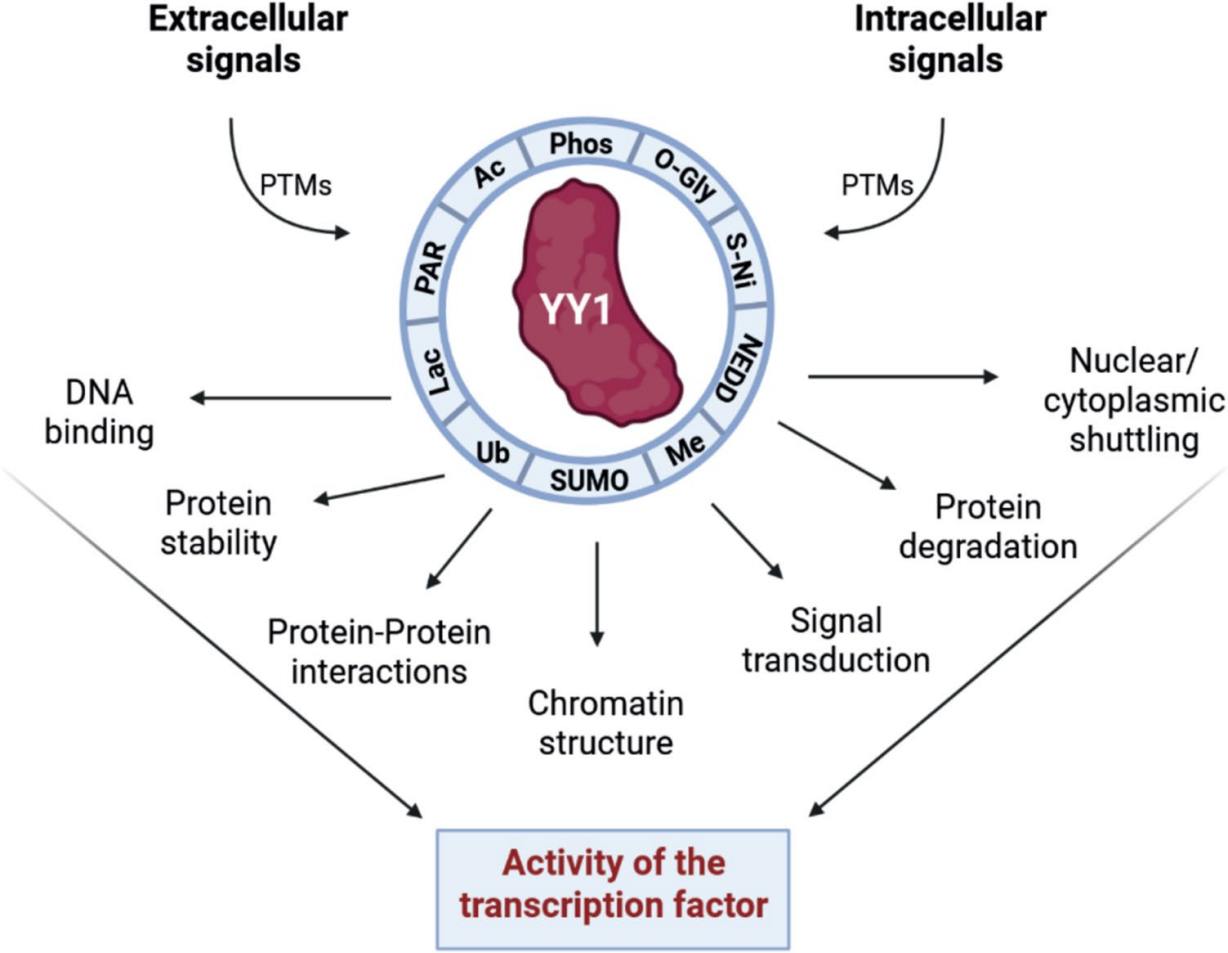
YY1 posttranslational modifications. Different extracellular and intracellular inputs result in YY1 posttranslational changes that ultimately modified its activity through structural and functional changes. *Ac* Acetylation, *Phos* Phosphorylation, *O-Gly* O-GlcNAcylation, *S-Ni* S-Nitrosylation, *NEDD* Neddylation, *Me* Methylation, *SUMO* Sumoylation, *Ub* Ubiquitination, *Lac* Lactylation, *PAR* poly(ADP-ribosyl)ation

Given the lack of evidence that rule the active or repressive activity of YY1, it has been suggested that YY1 posttranslational modifications (PTMs) can ultimately favor protein–protein interactions with other *trans*- elements, impacting in its subcellular localization, protein stability and activity [19, 71]. The interplay among different YY1 PTMs that might regulate its function and activity is depicted in Table 1 and Fig. 2 [41].

**Table 1:** YY1 Post-Translational Modifications (PTMs)

PTM	Functional outcome	PTM site	Enzyme	References
Phosphorylation (Phos)	YY1 decrease DNA-binding Modulation of YY1 sequence recognition Protein–Protein interactions Prevention of YY1 cleavage by caspases	Thr 348, 378 Tyr 383 Ser 365 Thr 39 Ser 184, 180 Thr 30, Ser 365 Ser 118	– Src Kinases Aurora A Plk1 Aurora B mTORC1 signalling CK2α	[68, 73–81]
Acetylation (Ac)	Higher transcriptional repression activity YY1 binding to enhancer and promoter regions (Transcription activation)	C-terminal domain	PCAF and p300	[84, 85]
Ubiquitination (Ub)	YY1 degradation	–	E3 ubiquitin ligases (Neddd4, SMURF2)	[86, 87]
SUMOylation (SUMO)	YY1 stability Modulation of its transcriptional activity (Enhancing or repressing)	Lys 288	SUMO E3 Ligase (PIASy) SUMO conjugating enzyme E2 (Ubc9)	[83]
Methylation (Me)	Increase YY1 DNA-binding	Lys 173, Lys 411	SET7/9	[88]
S-Nitrosylation (S-Ni)	Inhibition of YY1 DNA-binding	–	NO reaction with Zn^2^⁺	[89, 90]
O-linked N-acetylglucosaminylation (O-Gly)	Specific protein–protein interactions Enhancement of YY1 stability	–	O-GlcNAc transferase	[91]
Poly(ADP-ribosyl)ation (PARylation)	Enhancement of PARP-1 activity Decrease of YY1 DNA-binding activity	–	PARP-1	[94, 95]
Lactylation (Lac)	YY1 gene modulation under hypoxic conditions	Lys 183	p300	[98]

*Thr* Threonine, *Ser* Serine, *Tyr* Tyrosine, *Lys* Lysine, *Plk1* Polo-like kinase 1, *CK2α* Casein Kinase II α, *PCAF* p300-CBP Associated Factor, *PARP-1* Poly(ADP-ribose)-polymerase-1, *mTORC1* Mechanistic Target of Rapamycin, *Neddd4* Neuronal Precursor Cell Developmentally Down-regulated 4, *SMURF2* SMAD Ubiquitination Regulatory Factor 2, *PIASy* Protein Inhibitor of Activated STAT Y, *Ubc9* Ubiquitin-conjugating enzyme 9, *SET7/9* SET domain-containing protein 7/9

The most extensively studied YY1 PTM is phosphorylation* (phos)* [41, 72]. YY1 is stably phosphorylated regardless of the cell cycle and differentiation state, this PTM is associated most likely to its DNA-binding ability [22]. I*n vitro* studies have shown that YY1 phosphorylation on activating sites increases its DNA binding activity, and if phosphorylation occurs on YY1 repressing sites it will decrease its DNA binding activity [73]. However, punctual and specific phosphorylation events have different outcomes, for instance phosphorylation on threonine 348 and threonine 378 sequesters YY1 in the cytoplasm and reduces its DNA-binding activity [68]. During G2/M stage of the cell cycle, Polo-like kinase 1 (Plk1) and Aurora B kinase phosphorylate YY1 at threonine 39 (at the N-terminus activation domain) and at serine 184 and 180 (at the glycine/alanine (GA) rich region), respectively, affecting its specific DNA sequence recognition and protein–protein interactions [73, 74]. Aurora A kinase phosphorylates YY1 in serine 365 in the third zinc finger during mitosis, abolishing its DNA binding activity [75]. The activation of the Src family of non-receptor tyrosine kinases via epidermal growth factor receptor (EGFR) signaling, targets tyrosine 383 located at the very start of the fourth zinc finger, interfering with its binding activity and affinity for other cofactors [76]. Casein Kinase II α (CK2α) phosphorylates YY1 at serine 118 (at the transactivation domain), preventing its cleavage by caspase 7 during apoptosis [65, 77, 78], inhibition of CK2α decreases serine 118 phosphorylation and enhances YY1 cleavage, proving a regulation between YY1 and programmed cell death [77]. The Extracellular Signal-Regulated Kinases (ERK) pathway activation has been associated to YY1 phosphorylation favoring both transcriptional activation [79] and inhibition [80], although a direct ERK/YY1 has not been demonstrated.

Phosphorylation of YY1 might not only modulate its ability to bind DNA but also regulate its protein–protein interactions. The Mechanistic Target of Rapamycin (mTORC1) signaling pathway inhibits the interaction between YY1 and polycomb protein Pc2 through YY1 phosphorylation at threonine 30 and serine 365. Upon rapamycin treatment, YY1 is dephosphorylated promoting its interaction with Pc2 and recruited to the genome acting as a transcriptional repressor. Interestingly, this association was correlated to lysine 27 histone H3 methylation (H3K27) potentially mediated by the histone methyl transferase Ezh2 another polycomb protein recruited by YY1 [81]. Moreover, YY1 can physically interact with mTORC1 through its Raptor and the REPO domain [81, 82] suggesting that mTORC1 is capable to dock YY1 to other proteins as the polycomb corepressors [81].

Out of its 414 amino acids YY1 contains 32 lysines. Lysine is an important target for acetylation, sumoylation, ubiquitination, methylation and neddylation [83]. These modifications modulate its association to other proteins and therefore its activity [71]. For instance, *acetylation (Ac)* of the central glycine-lysine (GK) rich domain (aa 170–200) by p300-CBP associated factor (PCAF) and p300 is required for a more effective transcriptional repressor activity, consequently such modification is targeted by HDAC1and HDAC2 to remove it [84]. Acetylation of the C-terminal domain (aa 261–333) by PCAF reduces its DNA binding capability and it cannot be deacetylated, rather, this acetylation prompts YY1-HDACs association intimately related to histone H4 deacetylation [84]. Moreover, YY1 acetylation is required for DNA binding to enhancer and promoter regions simultaneously and activate transcription [85], giving a fine-tune transcriptional activity regulation.

YY1 is degraded by *ubiquitination (Ub)* by E3 ubiquitin ligases like the neuronal precursor cell developmentally down-regulated 4 (Nedd4) [86] and by SMAD ubiquitination regulatory factor 2 (SMURF2) [87]. In contrast, the SUMO E3 ligase, PIASy co-localizes with YY1 in the nucleus and *sumoylates (SUMO)* YY1 principally at lysine 288, increasing YY1 stability and modulating YY1 transcriptional activity differentially (enhancing and repressing the transcription of different genes) [83]. Moreover, SUMO conjugating enzyme E2, Ubc9, can also interact and sumoylate YY1 (in vitro) in absence of any SUMO E3 ligase, the outcome of this modification remains to be elucidated [83].

YY1 can also be methylated, it has been demonstrated that SET7/9 can *methylate (Me)* YY1 in vitro in at least one site in the amino terminal (Lys 173) and five sites in the carboxyl terminal (Lys 288, 305, 339, 341, 441), being lysine 411 and 173 methylation important to increase its DNA binding activity [88]. Interestingly, nitric oxide reacts with YY1 resulting in *S-nitrosylation (S-Ni)* of critical cysteine-thiol residues coordinated by Zn^2^⁺, leading to impairment of the zinc fingers function, this change leads to the inhibition of YY1 DNA-binding activity [89, 90].

In response to high glucose levels, YY1 can be modified by carbohydrate addition known as *O-*linked *N*-acetylglucosaminylation (*O-GlcNAcylation*) by O-GlcNAc transferase without affecting its DNA binding capability and regardless the cell differentiation status [91]. This modification is related to the heterodimerization with the retinoblastoma protein (Rb)-YY1 in which Rb inhibits the interaction of YY1 with DNA, blocking YY1-dependent transcription [2], YY1 *O*-GlcNAcylation dissociates it from Rb and allows it to bind DNA, demonstrating a physical and functional association between YY1 and Rb in cell cycle transitions [91]. It has been shown that O-GlcNAcylation of YY1 can also enhance YY1 stability [92]. The specific sites for this modification remain to be elucidated.

YY1 can physically interact with the nuclear enzyme poly(ADP-ribose) polymerase 1 (PARP-1) in vivo and in vitro, particularly after genotoxic insults [93]. The interaction between YY1 and PARP-1 depends on the transient YY1 poly(ADP-ribosyl)ation (*PAR* or *PARylation*) by PARP-1, such YY1 modification enhances the enzymatic activity PARP-1 and decreases YY1 DNA binding affinity, accelerating DNA repair and inducing transcriptional silencing [94, 95]. PARP-1 participates in the DNA repair process via the base excision repair pathway to rejoin nicked strands of DNA. The described mechanism between YY1 and PARP-1, seems to maintain cellular stability after DNA damage by repairing DNA while decreasing the expression of damaged genes, since YY1 regulates the expression of many genes involved in cellular proliferation [96]. A novel and very interesting PTM has emerged in recent years, *lactylation* (Lac), consisting in the addition of lactate, typically on lysines of different proteins including histones and is associated to gene expression stimulation [97]. Lactate is a metabolic bioproduct and an important energy source and commonly increased under hypoxic conditions. YY1 can be lactylated at lysine 183 by p300, giving a fine tune modulation on the expression of certain genes [98].

## Transcriptional Regulation Mechanisms by YY1

**Fig. 3:**
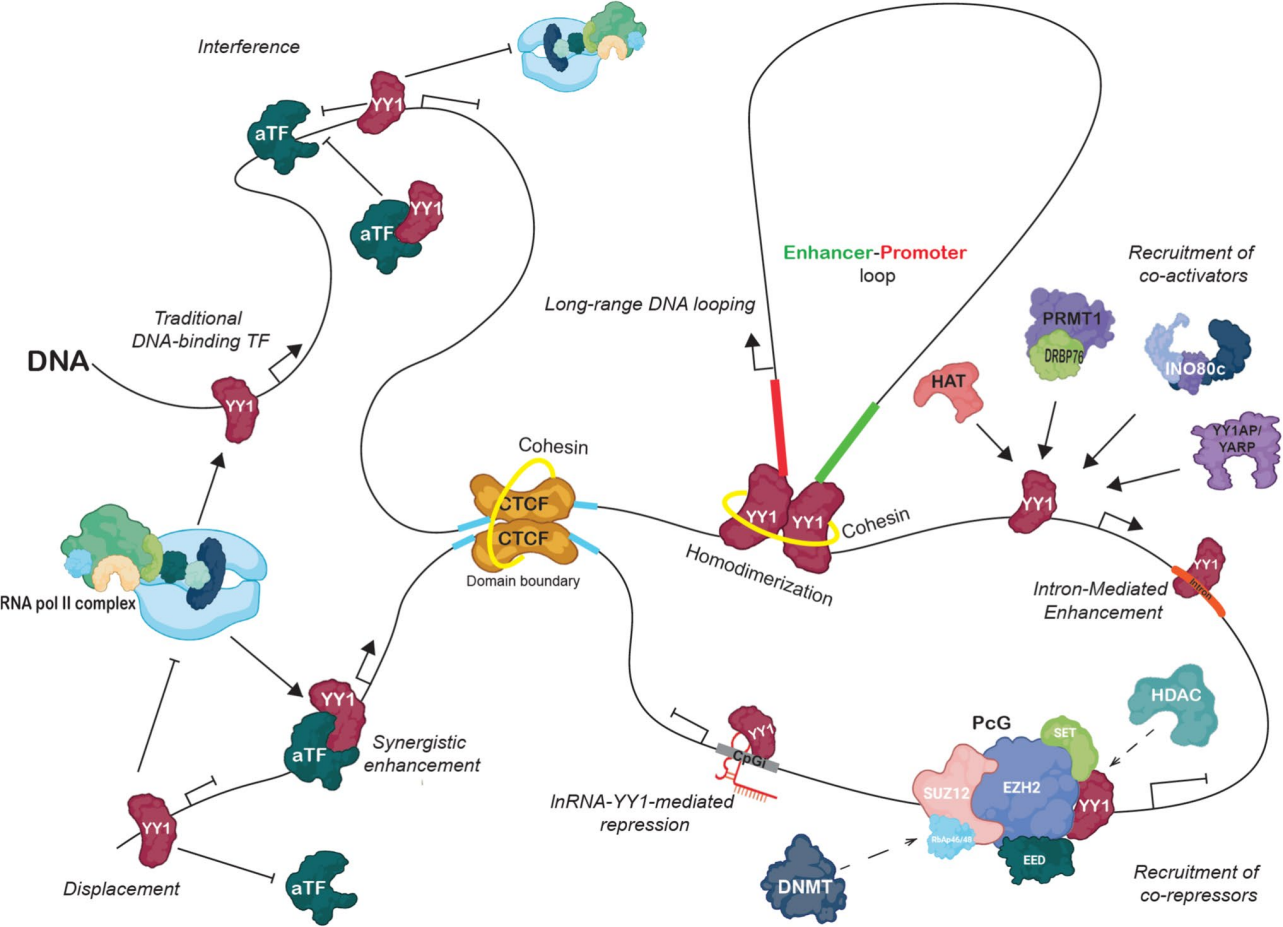
Transcriptional regulation mechanisms of YY1. YY1 can activate gene transcription directly by *traditional DNA-binding* to gene promoters or can bind to other transcription factors to stimulate transcription activation in a *synergistic enhancement*. Another way of activation is through protein–protein interactions with co-activators that are usually chromatin modifiers that regulate chromatin accessibility. YY1 orchestrates chromatin rearrangements via enhancer-promoter loops within larger topologically associated domains delimited by other structural proteins such as CTCF and cohesin. *Intron-mediated enhancement* results from the YY1 positioning in an intron near the transcription site potentiating transcription. The gene repression mechanism can result from *interference* or *displacement* of other transcription factors or the transcriptional machinery. Also, the *recruitment of co-repressors* that could modulate chromatin rearrangements to a close conformation, or with DNA modifiers that repress transcription such as DNA-methyltransferases. Figure done with BioRender

A plethora of genes that contain potential YY1 binding sites within their promoters have been reported and an important number of genes have proven to be regulated by it, including p53, c-Myc, c-Fos, erbb2, cdc6, histone H3.2 and H4 genes [99]. YY1 acts through its consensus *cis* recognition sequence: 5’-CCG(A/C)CATNTT-3’ [11, 16]. However, its molecular activity will not always lie on its physical interaction with its recognition sequence in the DNA, but in its interaction with various epigenetic writers and erasers, increasing the complexity of gene expression regulation (Fig. 3).

The classical gene regulation exerted by YY1 can be divided in different stages, beginning with the binding of YY1 to its consensus binding site on DNA, then, YY1 can recruit other TF and cofactors to regulate direct gene expression, and last, YY1 can recruit protein modifiers to prompt posttranslational modifications on histone and non-histone proteins associated with YY1 to the YY1-targeted DNA regions, modifying chromatin structure [41]. YY1 can also direct physical modifications on DNA structure and lead transcriptional regulation [100]. Moreover, YY1 can act as a co-factor of other TFs enhancing the association of the TF to its response element, where YY1 DNA binding activity is dispensable (Fig. 3) [18, 101, 102]. Ultimately, the active or repressive characteristic of YY1 depends on different interactions with cell type-specific factors.

### YY1 Mechanisms of Gene Activation and Gene Silencing

YY1 can act as a traditional DNA-binding factor (transcriptional initiator), in which its positioning on its DNA element at promoter regions triggers the positioning of the transcriptional machinery [19, 41, 103]. Protein–protein interactions of YY1 can occur with other basal or specific activating transcription factors (aTF), just like YY1 can interact directly with Sp1, TATA-binding protein (TBP), TBP-associated factors, transcription factor IIB, promoting a synergistic enhancement of transcription initiation [22, 103–105]. For instance, in response to endoplasmic reticulum (ER) stress, ATF6 induces the expression of glucose-regulated protein genes that encode ER chaperones, this expression is greater when ATF6 binds to YY1, enhancing the transcriptional activity to induce such genes [106].

The positioning of YY1 on its DNA-binding elements can recruit transcriptional co-activators which can be divided into three classes: (a) ***Adapters*** that initiate the recruitment of the transcriptional machinery, (b) ***Chromatin-remodeling enzymes*** and (c) ***Mediators*** that modify or interact with other TFs, inducing a reaction at the promoter. For example, in nuclear YY1-rich puncta, coactivators such as EP300, BRD4, MED1 and active RNA polymerase II can be easily detected [53, 103]. Likewise, YY1 can recruit the protein arginine methyltransferase 1 (PRMT1) to YY1-activated promoters, via the accessory protein DRBP76 acting as a scaffold, resulting in histone H4 Arg33 methylation, leading to a stable transcriptional active chromatin conformation [107]. Other proteins like YY1 associated factor 1 (YY1AP) and its homolog YARP, or the YY1-assoaciated factor 2 (YAF-2), are tethered directly to promoters as YY1 cofactors boosting transcription [108, 109]. Thus far, it is not well understood if different YY1 protein–protein interactions are mutually exclusive or synergistic. Interestingly, YY1 forms part of a bigger chromatin remodeling complex, the human INO80/YY1 chromatin remodeling complex composed of 15 subunits that catalyzes ATP-dependent sliding of nucleosomes along DNA, thereby regulating gene expression through chromatin structure alterations [5, 110]. YY1 can recruit the INO80 complex to YY1-activated genes, such as CDC6, GRP78, BCCIP, functioning as an essential coactivator. The current hypothesis suggests that YY1 binding to its DNA binding element requires INO80, implying that YY1 uses the INO80 complex not just to activate transcription but also to gain access to the targeted promoters [110, 111]. There is a growing list of YY1 interactions and cooperativity with other mediators or TFs, that ultimately reflects the plethora of cellular processes in which YY1 is involved and it will essentially depend on the cellular context, cell type and tissue [112, 113].

In recent years, it has been demonstrated that YY1 has the ability to homodimerize, bringing together two distant elements on the genome inducing a DNA bending, moreover, the binding of YY1 with accessory proteins that modify histones can easily promote chromatin rearrangements that will repress or activate transcription in a chromatin accessibility-dependent manner [19, 61]. The homodimerization of YY1 not only regulates the expression of proximal genes, but it can also control the expression of distal genes through *long-range DNA looping*, acting together with chromatin structural proteins such as CCCTC binding factor (CTCF) and cohesin, insulating topologically associating domains (TADs) [61]. YY1 binds Guanine-quadruplex structures, and by these means homodimerize occupying two long distance elements as active enhancers and promoter-proximal elements, bridging promoters and enhancers, and coordinating interactions of coactivators and chromatin elements to regulate gene expression [60, 61, 110]. YY1 is able to bind DNA elements within intron sequences, downstream or near the transcription initiation site acting as a classical transcriptional enhancer and potentially producing alternative mRNAs, thereby increasing mRNA accumulation, in a mechanism named *intron-mediated enhancement* (IME) [114, 115]. This intron-dependent effect adds a further layer of regulation to YY1-regulated gene expression (For further information about this mechanism see [116].

YY1 can exert transcriptional repression through various mechanisms that end in the downregulation of a specific gene. In an open/active chromatin context, YY1 can *displace* an aTF whose DNA binding element might be overlapping a YY1 binding site, competing alternatively for the occupancy on such promoter by being mutually exclusive [19]. YY1 can also interfere with other aTF by binding distally (upstream or downstream) in the same promoter, repressing the activity of the aTF or directly by inhibiting the positioning of the transcriptional machinery, in an *interference* mechanism [41]. In this mechanism there are two alternative interference types, one involves DNA binding whereas the other is DNA binding-independent. This is the case when a certain aTF is directed to its DNA-binding element but is intercepted by YY1 through protein–protein interactions, inhibiting the aTF binding to the promoter (Fig. 3) [117]. Protein–protein interactions also modulate YY1 activity, c-Myc is known to bind YY1 inhibiting both its repression and activation functions, hence, c-Myc is in fact, a YY1 modulator [18]. Likewise, YY1 interacts with the conserved N-terminal Mad homology 1 domain of Smad proteins (Smad4, Smad1, Smad2 and Smad3) which transduce TGF-beta and bone morphogenic protein (BMP) signals to regulate cell growth and differentiation. YY1 inhibits binding of Smads to their cognate DNA elements, and regulates cell differentiation induced by TGF-beta superfamily pathways [4].

YY1 transcriptional repression also depends on protein–protein interactions with co-repressors and their recruitment to specific target locus in the genome. YY1 interaction with co-repressors is commonly associated with changes in chromatin remodeling [71]. The Polycomb group (PcG) proteins are modifying chromatin enzymes, that downregulate epigenetically gene expression, these proteins reside in two complexes: polycomb repressive complex 1 (PRC1) and 2 (PRC2) [118]. The gene silencing activity of PRC1 is based mainly in the activity of an E3 ubiquitin (Ub) ligase (RING1a-1b) that monoubiquitinates lysine 119 at the C-terminal tail of histone H2A interfering with RNA polymerase, in contrast PRC2 gene silencing resides on its ability to di- or thrimethylate lysine 27 of histone H3 via the enhancer of Zeste homolog 2 (EZH2) [119, 120]. Both mechanisms lead to chromatin compaction reducing the accessibility to transcriptional machinery, transcription factors and ATP-dependent chromatin -remodeling machineries [121]. The mechanisms of PcG complex recruitment has been extensively studied, this is in part due to the large size and inconsistent sequence homology of characterized polycomb response elements (PREs) and that PcG proteins cannot specifically bind to DNA sequences, nevertheless, the PRE contain YY1 core consensus binding sites [122, 123]. YY1 is the mammal homolog of Pleiohomeotic (Pho), member of PcG in *Drosophila* [124], and it can recruit PcG proteins to DNA [125] through its REPO domain (REcruitment of POlycomb, REPO) (YY1 residues 201–226) (Fig. 1) by the binding of the PcG protein Yaf2 which serves as a bridging protein between PcG and YY1/DNA [47, 48]. YY1 contributes to histone methylation by recruiting EZH2 to lysine 27 of histone H3 [126], while stablishing and maintain gene repression. YY1 can also physically interact with the Supressor of zeste 12 (SUZ12) a component of the PRC2, this interaction acts as a mediator to recruit the PcG proteins and DNA methyltransferases (DNMTs) to participate in gene silencing processes [127]. PRC2 can function as a recruiting platform for DNMTs, leading to DNA methylation, upgrading the transcriptional repression.

Importantly, the YY1 interaction with DNMTs has been proven [128, 129]. DNMT1 is the maintenance enzyme, it catalyzes the inheritance of methylation patterns in replicating cells, copying the pre-existing methylation pattern in the hemi-methylated strand onto the daughter new DNA strand. YY1-DNMT1 interaction proves its importance in DNA methylation inheritance [128]. On the other hand, the DNMT3 family includes DNMT3A and DNMT3B that catalyze the de novo methylation participating in the establishment of methylation patterns during development, it should be noted that these enzymes also methylate specific locus or genes in a direct manner. Since DNMTs do not have established DNA-binding elements, the role of anchorage to site-specific sites must be mediated through a mechanism involving transcription factors. DNMT3A and DNTM3B can interact with YY1 and potentially act as cofactors to silence YY1-targeted genes through DNA methylation [129].

As typical chromatin modulators, HDACs, can also be recruited by YY1 to represses transcription [130]. Furthermore, YY1 can also interact with lncRNAs to downregulate transcription. For example, the lncRNa Sox2ot interacts physically with YY1, likely with cofactors, leading this assemble to CpG islands at Sox2 promoter thereby downregulating its expression [131]. This supports the general archetype of lncRNA functioning as scaffold for protein recruitment [131].

The already mentioned mechanisms of YY1 to modulate gene expression may operate additively rather than exclusively, emphasizing that PTMs that can also modulate YY1 interactions with other proteins or modify its DNA binding ability.

### YY1 in Neurodevelopment

**Fig. 4:**
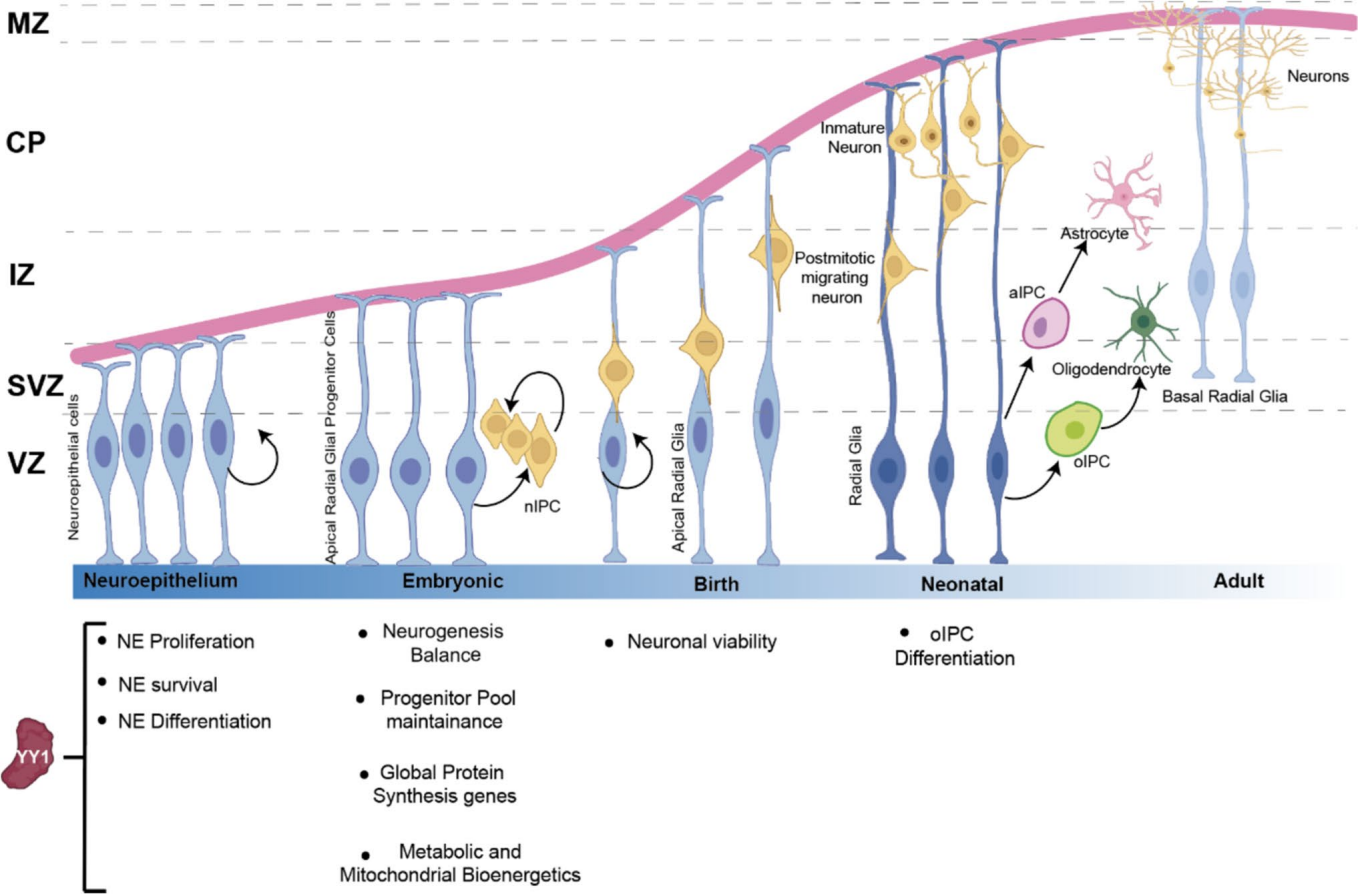
YY1 during neurodevelopment. YY1 is an important player on early stages of neurodevelopment. YY1 regulates survival, proliferation and differentiation by regulating apoptosis and key differentiation genes like SOX2 and REST. YY1-regulated genes are involved in metabolic pathways and protein translation, influencing the cell cycle machinery in neuroepithelial cells (NE) and neural progenitor cells. The decrease dependency on YY1 during neurodevelopment is given by the diminished biosynthetic demand and a decreased proliferation rate. *VZ* Ventricular Zone, *SVZ* Subventricular Zone, *IZ* Intermediate Zone, *CP* Cortical Plate, *MZ* Marginal Zone, *nIPC* neural Intermediate Progenitor Cell, *oIPC* oligodendrocyte Intermediate Progenitor Cell, *aIPC* Astrocytic Intermediate Progenitor Cell. Figure elaborated in BioRender

In vertebrates, neurons and glial cells derive from neuroepithelial cells (NECs) of the neural plate and subsequently form the pseudostratified epithelium that constitutes the early neural tube during neurodevelopment [132]. NECs are polarized cells positioned along their apical-basal axis spanning the width of the neuroepithelium (Fig. 4) [132]. NECs are also known as Neural Progenitor Cells (NPC), at the first round of neurogenesis, NECs are transformed into apical radial glia (aRG). Radial glial exhibit a characteristic bipolar morphology, with one end-foot on the ventricular surface and a radial process that extends to the pia [133], having cellular signatures that differs from NECs like cytoplasmic glycogen granules, expression of glial fibrillary acidic protein (GFAP), the astrocyte-specific glutamate transporter (GLAST), brain lipid-binding protein (BLBP) and Tenascin C (TN-C) [134]. While being a distinct cell type from NECs, aRG retain some neuroepithelial character, such as the expression of nestin. Both NECs and aRG undergo symmetric divisions to expand their cellular pools (self-renewal), and asymmetric divisions to give rise to intermediate progenitors, basal progenitors or postmitotic neurons [132, 135]. Given its features, radial glia cells (RG) are now also known as neural stem cells (NSC) that give rise to three major cell types in a temporally defined sequence, neurons appear first, followed by astrocytes and then oligodendrocytes [136]. Once differentiated, neurons migrate to form layers in an inside-out manner populating the outer layers, maintaining the newborn neurons closer to the inner cortex surface [133, 137]. In this step, RG functions as a scaffolding structure for the migration of postmitotic neurons until these cells reach their final destination. Such processes must have a fine-tune regulation in timing and differentiation [132]. The first stage in development of the CNS is solely neurogenic, this period is promoted by pro-neurogenic factors such as neurogenin 1, which simultaneously suppresses the gliogenic rout. Astrogliogenesis occurs in mid-late embryonic stages by the expression of astroglial genes determined by different transcription factors such as the nuclear factor I-A (NFIA), SOX9, Notch signaling and JAK/STAT cascade [138, 139]. Nevertheless, the gross of astrocyte population in the CNS arises during the second and third postnatal weeks, increasing from few million cells to hundred million cells [140]. Astrocytes and oligodendrocytes do not always come directly from the division of NSC but can come from precursors with more restricted potential (fate-restricted progenitors): the intermediate progenitor cells (IPC). IPCs can generate neurons (nIPCs) or generate glial cells, including oligodendrocytes (oIPCs) or astrocytes (aIPCs) (Fig. 4) [133, 135]. Later in neurodevelopment, RG cells in most brain regions are transformed into astrocytes when neuronal generation and migration are completed. However, in some other brain regions radial glia persists in the adult CNS as basal RG (bearing a basal process that contacts the basal lamina but lacking an apical process that contacts the ventricle), such as Bergmann glia in the cerebellum, Müller glia in the retina and some persisting radial glia in the dentate gyrus of the hippocampus [135]. Interestingly, in adult CNS RG are mitotically active cells, and considered as basal progenitor cells [141, 142].

During embryogenesis, the CNS complex cytoarchitecture has a perfect timing and fine-tuned regulation. Moreover, given the intricate and vast array YY1-regulated genes on different cellular processes, its participation during the CNS embryonic development is well documented [3]. Studies in mice lacking YY1 gene (homozygous deletion) display a peri-implantation lethality and embryonic defects, not surviving. YY1 heterozygote deletion results in growth retardation and neurulation defects. The abnormal embryos show exencephaly, asymmetric structure and pseudo-ventricles formation. [1]. Similar results were observed in *Xenopus laevis* using a different YY1-silencing approach [143]. The haploinsufficiency of YY1 in humans causes Gabriele-de Vries syndrome, and as a result white matter abnormalities delay myelination and enlarge brain ventricles, this is clinically characterized by psychomotor delay and intellectual disability together with many comorbidities (e.g., craniofacial dysmorphisms, intra-uterine growth restriction and behavioral alterations) [144].

During the embryonic stage, neural crest (NC) harbors the stem cell population that gives rise to different cell types that include neurons and glia. Notably, YY1 is identified as a master regulator of the NC transcriptional program [145]. YY1 is crucial during CNS early stages of development by regulating the proliferation and survival of neural progenitor cells (NPC) with no changes in differentiation patterns [146]. In NPC, YY1 controls the expression of metabolic, mitochondrial bioenergetic and global protein synthesis genes [146]. During early embryonic neuroepithelial development, conditional YY1 knock-out mice in the mid-hindbrain region resulted in cerebellar agenesis and dorsal midbrain hypoplasia by disrupting cell cycle, Wnt1 signaling, and p53-induced cell death of neuroepithelial cells [147]. These abnormalities are the result of YY1 deletion, since Wnt1 transcriptional activation requires YY1 binding to the Wnt1 promoter region [147]. On a regular basis, YY1 disrupts p53-p300 interaction and thus p300-dependent acetylation reducing p53 stability, therefore, YY1 absence leads to p53 accumulation [148]. Moreover, when YY1 and p53 are knocked-out simultaneously, NPC survive [146]. YY1 can also induce hypermethylation of the FUZ promoter, the planar cell polarity effector gene that is an important apoptosis factor in neuronal development [149]. In summary, developmental YY1-dependence relies on apoptosis inhibition, facilitating NPC survival.

In NPC, Sox2 is a pluripotency gene in charge of maintaining their stemness whereas in postmitotic cells, Sox2 is downregulated allowing NPC differentiation into neurons [150]. Interestingly, a highly conserved lncRNA overlaps Sox2, it is named Sox2 overlapping transcript (Sox2ot) which binds to YY1, potentially recruiting YY1 cofactors, to the CpG islands at Sox2 locus repressing its transcription [131]. Hence, higher Sox2ot expression represses Sox2 gene expression in negative feedback. YY1 knock out increases NPC population and decreases neurons [131]. Intriguingly, YY1 is also a positive regulator of the Repressor Element 1-Silencing Transcription factor (REST), which plays a role in restricting the expression of neuronal genes in non-neuronal cells, suggesting that lack of YY1 might induce neuronal differentiation [151]. Therefore, YY1 is important player in the maintenance of the progenitor pool and the neurogenesis balance, but also, it is also involved in the fine tuning of synaptic establishment.

Later in differentiation stages, YY1 is a critical regulator of oligodendrocyte progenitor differentiation, by interacting and recruiting HDAC1 to the promoters of inhibitors of myelin gene expression (Tcf4, Id4 and Sox2), hence, YY1 acts as a lineage-specific repressor. However, YY1 expression is necessary but not sufficient for oligodendrocyte progenitor differentiation [152, 153].

### Glial Cells in the CNS

Glial cells also known as neuroglia, are critical components of the CNS, due to compulsive presence for a neuronal proper functioning. Glial account for approximately 20 to 50% of the total number of brain cells [154, 155], in fact in some brain regions astrocytes can outnumber neurons, thus, in the last decades these cells have entered the research landscape to understand the physiopathology and pathogenesis of neurological and neurodegenerative diseases. Glial cells regulate much of the microenvironment surrounding neurons regulating the blood brain barrier [156], maintaining ion balance [157], participating in neurotransmitters recycling [157], providing metabolic supply [157], strengthening and pruning of synapses [158], scavenging debris and immune functions [158], and during development as neural precursors, regulating the neurogenic niche and guiding neuron migration [159].

Only recently it is that glial cells have been recognized as key components of the fine-tuning of brain development, given their known participation in the establishment of neural circuitry by regulating synaptogenesis [160], enhancing synaptic plasticity [161]. Accordingly, a small variation in glial cell density leads to a variation in the amount of synapses per volume of parenchyma, independently of neuronal numbers [154]. Neuroglia and neurons communicate through a bidirectional exchange of ions, neurotransmitters, metabolites, cell adhesion molecules and extracellular vesicles. The intercellular diffusion of these chemical messengers, maintain homeostasis and regulate synaptic transmission in health and disease [162].

Glial cells can be divided according to their embryonic origin in *macroglia* from the neuroectoderm and *microglia* arising from peripheral mesodermal tissue. In the CNS, macroglia is composed of astrocytes, oligodendrocytes, radial glia, ependymal cells and even NG2-Glia cells. While, microglia are regarded as the tissue-resident macrophages of the CNS, these cells are part of the innate immune response and are also involved in clearing cellular debris and maintaining tissue homeostasis [163]. An interesting feature of glial cells is their heterogeneity and plasticity which enables them to alter their phenotype in response to the micro environment. Moreover, their strategic positioning between vasculature and neurons, prompts them to mediate the exchange of molecules between these compartments [162, 164].

In the adult rodent brain, YY1 is ubiquitously expressed albeit at different levels depending on the brain region and cell type, thus YY1 is found predominantly in the olfactory bulb, cerebellar cortex, hippocampus, cerebral cortex, wall of the lateral ventricle and the rostral migratory stream. It is expressed intensively in neurons, oligodendrocytes and microglial cells [165]. In the cerebellum, even when YY1 is dispensable for initial stages of astrocytes development, it regulates subtype-specific gene expression during astrocyte maturation and this regulation helps to maintain a mature astrocyte phenotype [166].

#### Physiological Importance of YY1 in Astrocytes

Astrocytes account for the most extensive glial fraction, occupying a large fraction of the adult brain volume, its ultrastructure is an enormously elaborated network of fine processes. In rat hippocampus, a single astrocyte can reach and wrap around 140 000 synapses [167], whereas in human brain, an astrocyte is estimated to contact over a million synapses [168]. Among excitatory synapses the number of synapses contacted (or ensheathed) by astrocytes varies from the different brain regions (striatum ~ 80%, hippocampus ~ 50–90% and in cerebellum ~ 60–90% of the synapses), and such contacts are dynamic and constantly changed by neuronal activity, in such a way that an increase neuronal activity leads to an increase in the extent of astrocyte coverage of dendritic spines together with increase in the number of spines contacted by astrocytes processes [169–172].

Glial fibrillary acidic protein (GFAP) is a signature marker of astroglia cell population [173]. GFAP is a structural cytoskeletal protein (Intermediate filament protein) and is involved in the anchoring and trafficking of membrane proteins within the astrocytes. GFAP has been typically used to identify astrocytes, but since not all astrocytes express GFAP and not all cells in the CNS that express GFAP are astrocytes, the use of other markers such as S100b, glutamine synthetase or Aldh1L1 are more accurate to phenotype them among a complex heterogeneous astrocytic population [174].

To tackle *astrocytic YY1* relevance in brain physiology, recent studies have used glial fibrillary acidic protein (GFAP)-controlled YY1 conditional knockout mice (YY1 cKO) model [166, 175]. YY1 cKO mice showed abnormal phenotype, movement deficits, and cognitive dysfunction. This outcome was the result of altered expression of genes associated with proliferation and differentiation, p53/caspase apoptotic pathways, increased oxidative stress, and inflammation induction, together with inflammatory signaling including NF-κB, STAT and IRF in cortex, midbrain and cerebellum [175]. During later stages of postnatal cerebellar development, deletion of YY1 in astrocytes leads to impaired astrocytic maturation, apoptosis and increased expression of inflammatory genes, resulting in motor deficits in mice by inducing Bergmann gliosis together with loss of GFAP expression in velate and fibrous cerebellar astrocytes impairing locomotor activity, coordination, and memory function [166].

Glutamatergic neurotransmission accounts for a considerable part of total energy requirement related to neural signaling processes. Glutamate (Glu) is the major excitatory neurotransmitter in the CNS and given the lack of extracellular enzymes that can metabolize Glu to halt neurotransmission, makes Glu removal compulsory, this is carried out by plasma membrane Glu transporters [176]. The gross of Glu uptake (~ 80–90%) is mediated through glial Glu transporters, properly named Excitatory Amino Acid Transporters (EAATs). EAATs prevent an uncontrolled and persistent activation of Glu receptors and control the Glu diffusion to peri- and extra synaptic receptors. Moreover, Glu uptake enables the recycling of the neurotransmitter, conferring protection against hyperexcitability and excitotoxicity resulting from Glu spillover [176, 177]. Glial cells mainly express two glutamate transporters, the excitatory amino acid transporter 1 (EAAT1, also known as GLAST for Glutamate/Aspartate Transporter) and the excitatory amino acid transporter 2 (EAAT2, also known as GLT-1 for Glutamate transporter 1) [176]. Glu transporters dysfunction is linked to various neurological disorders such as stroke, amyotrophic lateral sclerosis (ALS), Alzheimer's disease (AD), Parkinson’s disease (PD) and Huntington’s disease (HD) [178]. EAAT1 and EAAT2 have differential expression around the CNS, while EAAT1 is enriched in cerebellar Bergmann glial cells (BGC), EAAT2 is highly expressed in astrocytes around the cortex and hippocampus [176]. Interestingly, extensive studies have demonstrated that both transporters are under YY1 transcriptional regulation, either increasing or decreasing its transcription under certain conditions and stimulus [179–181]. In chick BGC, Glu treatment increases YY1 binding to the *glast* promoter, resulting in its downregulation, consequently decreasing Glu uptake [181]. In cortical astrocytes, Glu exerts the opposite effect on *glast* expression by increasing rather than decreasing its expression [182].

Environmental exposure to xenobiotics accounts for several CNS dysfunctions by altering neurons and glia. Given the specific glial plasticity and location at the blood vessels in an interface with neurons, these cells participate actively in the integration and transmission of peripheral information to neuronal networks in the brain [183]. Moreover, neurons low capacity to endure adversities turn them into the most vulnerable target in the CNS, in that sense, glia cells are the responsive cellular elements to counteract injury and neurotoxicant effects, by undergoing cellular, molecular and functional changes referred to as reactive astrogliosis [184]. Chronic exposure to manganese (Mn) is a major cause of *manganism*, a neurodegenerative disorder clinically similar to Parkinson’s disease [185]. Mn preferentially accumulates in the basal ganglia particularly in the *substantia nigra* (SN), causing dopaminergic neuronal injury that results in behavioral deficits and impaired motor coordination [186]. Mn-induced excitotoxic neuronal injury, is associated with astrocytic Glu transporters impairment. Mn excitotoxic effect is mediated through N-methyl-D-aspartate (NMDA) receptors [187]. Mn exposure reduces EAAT1/GLAST and EAAT2/GLT-1 mRNA and protein levels [179, 180]. Mn increases ROS levels and TNF-α production, this in turns increases the phosphorylation of the beta subunit of the IκB kinase (IKK) complex (IKK-β) promoting NF-κB (p65) translocation to the YY1 promoter, increasing its transcription and protein levels, then resulting in YY1 positioning on Glu transporters promoters [188]. Interestingly, NF-κB (p65) is a positive regulator of EAAT1/GLAST [179] and EAAT2/GLT-1 [189], but when both NF-κB (p65) and YY1 are co-expressed the stimulatory effect of NF-κB (p65) is abolished, suggesting that YY1 is dominant over NF-κB (p65) [179]. Moreover, TNF-α can downregulate EAAT2/GLT-1 expression independently of YY1 but through a distinct pathway also requiring NF-κB (p65) [188, 189]. When astrocytic YY1 is deleted in the *sustantia nigra*, the impairment in locomotor activity and coordination induced by Mn is attenuated, also mitigating GLAST and GLT-1 down-regulation [190]. This data points out that GLAST and GLT-1 play a central role in Mn-induced dopaminergic neurotoxicity. Furthermore, YY1 interacts with many epigenetic regulators, and in Glu transporters transcriptional regulation YY1 interacts with HDACs (Classes I and II) as co-repressors to target GLAST and GLT-1 genes [180, 191, 192].

Astrocytic YY1 can also act as an indirect modulator of Glu transporters and neuroprotection. YY1 regulates positively the expression of REST [151]. REST is associated to the activation genes related to neuroprotective effects, and in complex with CBP/p300 and CREB binds to EAAT2/GLT1 promoter to upregulate its transcription [193]. Transforming growth factor-alpha (TGF-α) is a growth factor associated to neuronal survival, differentiation and involved in neuroprotection. In cortical astrocytes, YY1 acts as a negative regulator of TGF-α by targeting directly the promoter region of the gene as proven by reporter activity assays, and this occurs in response to Mn exposure [194]. Other neurodegenerative diseases share astrocytic Glu transporters disfunction as a hallmark of the onset or progression of the pathology, and may involve potentially YY1 as a critical molecular regulator, for example HD disease [195], amyotrophic lateral sclerosis [196] and autism [197].

Astrocytic YY1 has proven to participate in the progression of other neurodegenerative pathologies like Alzheimer’s disease in which YY1 promotes the expression of the β-site amyloid precursor protein-cleaving enzyme 1 (BACE1), a prerequisite for β-amyloid peptides formation [198].

#### YY1 in Microglia

Microglia are the major mononuclear phagocytes in the CNS, during development these cells shape neural circuits by modulating the strength of synaptic transmission and sculpting neuronal synapses [199]. During CNS injury, microglia are responsible for phagocytosis and elimination of pathogens, dead cells, protein aggregates and soluble antigens [199]. Microglia sense physiological and pathological cues, scanning the microenvironment and in response, secrete cytokines and neurotrophic factors, promoting inflammation or repair [200]. Microglia possess features uncharacteristic of any other cell in the brain, like its high motility and dynamic structural responsiveness. Microglia can react to their surrounding environment changing their functional state into two distinct polarization profiles: a pro-inflammatory one (M1), responsible for cytokine, chemokine and neuroinflammation metabolites production, and an immunoregulatory one (M2), associated with neuroprotection and damage repair events. Although nowadays, this classification can be considered outdated, since microglia can exist in diverse, dynamic and multidimensional stages that depend on multiple levels (transcriptional, epigenetic, translational and metabolic), more than in a polarized stage [201].

The production and release of glial-derived inflammatory molecules in the brain has both functional and structural consequences. YY1 increases cytokine production in astrocytes [202], this leads to the activation of microglia, proving a tightly astrocyte-microglia cross-talk, playing critical roles in inflammatory responses in the CNS. This astrocyte-microglia communication is more important than expected, since, in astrocytic-YY1 cKO mice an increase microglial activation (as judged by a sustained Iba-labeling increase) was observed, together with elevated inflammatory cytokines and chemokines including TNF-α and CXCL10 [175]. In LPS stimulated microglia, YY1 is upregulated by the transactivation activity of NF-κB (p65), in turn YY1 associates with p65 and bind to the IL-6 promoter increasing its transcription, while in non-stimulated cells YY1 suppresses IL-6 transcription by interacting with HDAC1 [203]. Overall, it is evident that astrocytic YY1 is also essential in preventing oxidative stress and inflammation [175].

TREM2 is a type I transmembrane receptor expressed in microglia and is required for the regulation of the immune response and phagocytosis, TREM2 senses tissue damage and activates a robust immune remodeling [204]. YY1 binds to TREM2 minimal promoter augmenting its expression in microglia, enhancing the clearance of Aβ in an AD model, suppressing the immune response [205]. In a model of sepsis-associated encephalopathy, YY1 promotes microglia M2 polarization via TREM2 upregulation by interacting with miR-130a-3p promoter [206]. TREM2 microglial expression enhanced by YY1 can serve as a potential strategy for disease and diagnosis.

In the retina, an important and often forgotten part of the CNS, hypoxic conditions induce YY1 hyperlactylation in microglia, resulting in its activation, proliferation and migration abilities, worsening the inflammatory scenario of autoimmune uveitis. This was achieved through the upregulation of inflammatory genes (eg. STAT2, CCL5, IRF1, IDO1 and SEMA4D) once YY1 lactylation is promoted by p300, the identified writer of such modification [207]. Microglia contributes to angiogenesis in the brain and the retina [208]. Microglia YY1 lysine 183 p300-dependent lactylation upregulates Fibroblast Growth Factor 2 (FGF2) mRNA levels, promoting neovascularization in oxygen-induced retinopathy [98]. All these findings, provide new insights on YY1 role on microglia.

#### YY1 in the periphery

In the peripheral nervous system (PNS), Schwann cells are the supporting glial cells that enwrap axons and produce myelin, tightly compacted and insulating sheaths, required for rapid conduction of electrical impulses thereby proper functioning of the PNS [209]. Axon-Schwann cell interface triggers PNS myelination through neuregulins and Egr2. Egr2 (also known as Krox-20) is a zinc finger transcription factor that controls a set of genes required for completion of myelination, Egr2 is regulated by axonal contact and is induced as Schwann cells begin to myelinate [210]. Egr2 expression is regulated by soluble and membrane-bound neuregulins [211]. Neuregulins are axonal growth factors that bind to ErbB receptor tyrosine kinases expressed in Schwann cells initiating a signaling cascade essential for modulating the timing and abundance of myelin [212, 213]. However, in-between extracellular signals and the expression of several genes orchestrated by Egr2, exists an intricate network that involves YY1 phosphorylation by the MEK signaling pathway that leads to YY1 positioning on Egr2 promoter and thereby increasing Egr2 expression. Schwann cells that lack YY1 have low Egr2 levels and severe hypomyelination, such phenotype is rescued by Egr2 overexpression [79]. These findings suggest that YY1 is a key modulator of peripheral myelination by regulating Egr2 expression in response to neuregulin1. Loss of myelin in the PNS leads to axonal degeneration and loss of nervous communication, in fact, it is the cause of several peripheral neuropathies and the major cause of persistent clinical impairment [209].

### YY1 and Cancer in the Nervous System

The already detailed YY1 features places it in a myriad of cellular processes. It has been proven that YY1 can be acutely modulated in cancer cells and that it can regulate genes related to cell cycle, cell death and tumor metabolism opening the possibility to use it as a novel target for therapeutic interventions [214].

Gliomas are the most common primary brain tumors of the CNS that originate from glial cells and can be classified histologically according on the involved cell type (e.g.: astrocytomas, ependymomas, oligodendrogliomas or mixed gliomas/oligoastrocytomas), but more recently gliomas can be typified based on molecular and genetic markers, having different degrees of malignancy ranging from grade 1 tumors (i.e., pilocytic astrocytoma) to grade 4 tumors (i.e. glioblastoma) [215, 216]. Glioblastoma (GBM) is one of the most aggressive malignancies with worst prognosis and is the most frequent glioma, with a 5-year survival rate below 10% [216]. Given the heterogeneity of GBM (that is pediatric GBM highly biologically distinct from adult GBM) [217], large-scale profiling studies have suggested that glioblastoma comprise at least six molecular subgroups characterized by distinct mutations and DNA methylation profiles, hence, different transcriptomic patterns [218]. In this sense, YY1 is highly expressed in GBM cells and plays important features in cell survival. Recent studies have shown a YY1 upregulation during the progression of the disease, increasing from benign meningiomas to low-grade gliomas and reaching major levels in glioblastoma multiforme [219]. You et al., showed that in GBM, YY1 promotes the expression of the SUMO-specific protease 1 (SENP1) which deSUMOylates methyltransferase-like 3 (METTL3) that adenine-methylates (m6A) MYC mRNA augmenting its expression. Through the regulation of the SENP1/METTL3/MYC axis, YY1 promotes the self-renewal of GBM cells contributing to the maintenance of cancer cell population, promoting tumor growth and contributing to the progression of the disease [220]. The small nucleolar RNA host gene 17 (SNHG17), a lncRNA involved in cell progression and migration in several cancers, is upregulated by YY1 in GBM allowing the activation of the Wnt pathway by sponging miR-506-3p a direct negative regulator of catenin beta 1, extending its half-life and consequently facilitating glioma progression [221]. On the other hand, miR-218 can target YY1 directly in glioma cells, inhibiting its function and suppressing cell proliferation promoted by YY1 [33].

In human cortical astrocytes, YY1 is localized almost exclusively in the cytoplasm, while in glioblastoma cells, YY1 is entirely nuclear [202]. This appears to be the result of YY1 restriction to actively proliferating cells, since in non-dividing cells YY1 is sequestered in the cytoplasm by the retinoblastoma protein (Rb)[2]. In GBM, YY1 is upregulated and it binds to cytokine promoters, increasing cytokine mRNA levels. This upregulation is likely to be responsible for the YY1 interaction with RelB and p50, and is associated to an inflammatory microenvironment that enhances the cancer progression [202].

Considering that glial Glu transporters are key players in glutamatergic transmission in the brain, and that their impairment contribute to the pathogenesis multiple neurodegenerative processes, altered Glu transporters expression in GBM represents an important step in glioma-induced neurodegeneration. The oncogene, Astrocyte Elevated Gene-1 (AEG-1) favors gliomagenesis in the context of tumor growth and invasion. AEG-1 represses EAAT2 expression at a transcriptional level by inducing YY1 activity through CBP inhibition as a coactivator of the EAAT2 promoter. During malignant progression, AEG-1 gradually increases with the parallel decrease of EAAT2 expression, this leads to reduction of Glu uptake in glial cells triggering neuronal death due to augmented extracellular Glu levels [222].

Research has been intensively addressing gliomas to understand their molecular pathogenesis and advance in diagnostics and delineate high throughput therapeutic strategies that could improve the survival of the patients harboring such tumors.

## Concluding Remarks

The ubiquity of YY1, its expression during development, in progenitor and differentiated cells, the plethora of protein–protein interactions with a variety of proteins/enzymes and its participation on highly controlled cellular mechanisms, have positioned YY1 under the scope of researchers to understand its key role in physiological mechanism as in tumorigenic mechanisms. Moreover, there is growing evidence of the critical YY1 involvement on the epigenetic landscape of cell-specific locus in different cell types. The fine-tunning mechanisms governing YY1 participation in many biological functions are yet to be determined. YY1 complex interactome has been a growing object of study that would delineate its function as transcriptional repressor or activator in complex cells like glia.

For many years, glial cells were neglected as important key players in CNS physiology, they were rather considerer as just the “*glue*” of neurons in the intricate CNS. However, nowadays the relevance of these cells in many brain physiological functions has become evident. Astrocytes are more than passive supporters of neurons and participate actively in maintenance and regulation of synaptic transmission. In this review, we provide a general view of the most important characteristics of YY1 as a protein, transcriptional regulator and its involvement in glia physiology. YY1 is a key player in early stages of neurodevelopment as a transcription factor and as a chromatin structural protein facilitating gene-specific long-distance promoter-enhancer DNA interactions. YY1 is associated with proliferation, survival and differentiation of astrocytes. Also regulating neurotransmission and metabolic gene changes.

Overall, astrocytic YY1 is required for a proper regulation of brain physiology, however, it is still not clear what cues dictate its effect during health and disease, if the overexpression, disruption or accumulation are the events underlying its activity under certain conditions, or if a more sophisticated regulation is dictating YY1 activity.

## Data Availability

No datasets were generated or analysed during the current study.
